# Altered White Matter Integrity in Smokers Is Associated with Smoking Cessation Outcomes

**DOI:** 10.3389/fnhum.2017.00438

**Published:** 2017-08-30

**Authors:** Peiyu Huang, Zhujing Shen, Chao Wang, Wei Qian, Huan Zhang, Yihong Yang, Minming Zhang

**Affiliations:** ^1^Department of Radiology, The Second Affiliated Hospital, Zhejiang University School of Medicine Hangzhou, China; ^2^Key Laboratory of Biomedical Engineering of Ministry of Education, Zhejiang University Hangzhou, China; ^3^Neuroimaging Research Branch, National Institute on Drug Abuse, National Institutes of Health, Baltimore MD, United States

**Keywords:** smoking cessation, diffusion tensor imaging, orbitofrontal cortex, cerebellum, postcentral cortex

## Abstract

Smoking is a significant cause of preventable mortality worldwide. Understanding the neural mechanisms of nicotine addiction and smoking cessation may provide effective targets for developing treatment strategies. In the present study, we explored whether smokers have white matter alterations and whether these alterations are related to cessation outcomes and smoking behaviors. Sixty-six smokers and thirty-seven healthy non-smokers were enrolled. The participants underwent magnetic resonance imaging scans and smoking-related behavioral assessments. After a 12-week treatment with varenicline, 28 smokers succeeded in quitting smoking and 38 failed. Diffusion parameter maps were compared among the non-smokers, future quitters, and relapsers to identify white matter differences. We found that the future relapsers had significantly lower fractional anisotropy (FA) in the orbitofrontal area than non-smokers, and higher FA in the cerebellum than non-smokers and future quitters. The future quitters had significantly lower FA in the postcentral gyrus compared to non-smokers and future relapsers. Compared to non-smokers, pooled smokers had lower FA in bilateral orbitofrontal gyrus and left superior frontal gyrus. In addition, regression analysis showed that the left orbitofrontal FA was correlated with smoking-relevant behaviors. These results suggest that white matter alterations in smokers may contribute to the formation of aberrant brain circuits underlying smoking behaviors and are associated with future smoking cessation outcomes.

## Introduction

Cigarette smoking is one of the most important causes of preventable mortality worldwide, and has been related to increased risk of respiratory (Dogar et al., 2013) and cardiovascular diseases (Pope et al., 2011). Among smokers who are aware of these risks, 3 out of 4 individuals are interested in quitting smoking (Heydari et al., 2014). While many smokers are interested in quitting, the cessation rate remains low, ranging from 12.4% (Hou and Cai, 2014) to about 30% (Brose et al., 2014; Santos et al., 2015). It has been found that smokers have large individual differences in response to current treatments for nicotine dependence (McClernon et al., 2008; Hall et al., 2015). Exploring the neurobiology of nicotine addiction and individual differences in smoking cessation outcomes may help to identify effective targets for developing better treatment strategies.

Neuroimaging studies have contributed significantly to understanding the neurobiological mechanisms of nicotine addiction. Altered brain structural (Gallinat et al., 2006; Zhang et al., 2011a; Liao et al., 2012) and functional changes (Due et al., 2002; David et al., 2005; Franklin et al., 2007; Zhang et al., 2011b) have been reported in widespread brain areas, indicating that several neural networks (Weiland et al., 2015) are involved in the mechanism of nicotine addiction. White matter connections are the foundation of neural networks. Fiber integrity greatly influences the efficiency of brain networks. Previously, several studies have investigated white matter alterations in smokers. Increased fractional anisotropy (FA) in the corpus callosum and anterior limb of internal capsule have been frequently reported (Jacobsen et al., 2007; Lin et al., 2013; Savjani et al., 2014; Viswanath et al., 2015), whereas decreased FA has also been found in various areas including corpus callosum (Paul et al., 2008; Kochunov et al., 2013), bilateral fronto-parietal white matter (Liao et al., 2011), internal capsule and some other regions (Yu et al., 2016). Interestingly, it was suggested that FA in several brain regions may increase in adolescent smokers but declines with continued smoking in adulthood (Hudkins et al., 2012), suggesting the plasticity of white mater during development. However, whether white matter integrity is related to smoking cessation success remains unknown.

In the present study, we aimed to demonstrate white matter alterations in smokers, and to investigate whether baseline white matter integrity is associated with smoking behaviors and future cessation outcomes. We utilized diffusion tensor imaging and tract-based spatial statistics (TBSS) to reveal the white matter alterations, which were considered more reliable than traditional voxel-based white matter analysis (Smith et al., 2006; Kieseppä et al., 2010). As the frontal-striatal circuit is a major neural network related to addiction (Li et al., 2015), we also expect to find white matter alterations in these areas.

## Materials and Methods

### Subjects

Sixty-six smokers and 37 non-smokers were recruited in the Second Affiliated Hospital of Zhejiang University School of Medicine. All participants were male, Han-Chinese, and right-handed. Smokers reported smoking ≥ 10 cigarettes per day in the last 1 year and met the DSM-IV criteria for nicotine dependence. Non-smokers were defined as individuals who had smoked less than 20 cigarettes in their lifetime and none in the past 10 years. Subjects with a history of major illness, other substance abuse (besides nicotine), psychotropic medications, neurological and psychiatric diseases, and systemic diseases (i.e., diabetes, hypertension) were excluded. Further exclusion criteria for all subjects included MRI contraindications, like claustrophobia and metal implants. Demographic and smoking data were obtained from all participants. Nicotine dependence severity was assessed in smokers using the Fagerström test for nicotine dependence (FTND) (Heatherton et al., 1991). Exhaled carbon monoxide (CO) was measured by a Micro Smokerlyzer (Bedfont Scientific Ltd., Kent, UK) to confirm participants’ smoking status (smokers ≥ 10 ppm, non-smokers ≤ 6 ppm). Smokers were allowed to smoke as usual prior to scanning to avoid withdrawal symptoms during scanning. We also assessed the basic neuropsychiatric condition of the smokers using Mini-Mental State Examination (MMSE), Hamilton Rating Scale for Depression (HRSD), and Hamilton Anxiety Scale (HAMA). This study was carried out in accordance with the recommendations of human research guidelines of the Institutional Review Boards of the Second Affiliated Hospital of Zhejiang University School of Medicine, with written informed consent from all subjects. All subjects gave written informed consent in accordance with the Declaration of Helsinki. The protocol was approved by the Medical Ethic Committee of the Second Affiliated Hospital of Zhejiang University School of Medicine.

### Medication

After MRI scanning and behavioral assessments, all smokers received a standard 12-week smoking cessation treatment with varenicline, which is a nicotinic receptor partial agonist (Niaura et al., 2006). Participants started with a recommended varenicline dosage of 0.5 mg once daily for 3 days, increasing to 0.5 mg twice daily for days 4–7, and then to the maintenance dose of 1 mg twice daily. Weekly telephone calls were conducted to query their smoking status and to provide smoking cessation counseling for the duration of their study participation. Participants were asked to reduce their smoking rate by 50% or more from baseline by week 4 with further reduction to 75% or more from baseline by week 8. Participants who self-reported continuous abstinence for weeks 9–12 (cross-validated with an expired CO level ≤ 6 ppm) were considered to be successful quitters. Twenty-eight smokers succeeded in quitting in the end. The successful cessation rate was 42.4% in this study, which is similar to results of previous studies on varenicline treatment (Jorenby et al., 2006; Nides et al., 2006). According to the cessation results, we divided the smokers into the quitter group and relapser group. Technically, the quitter and relapser group are “future” quitter and relapser group, because at baseline when MRI scans were performed, the outcomes were unknown. Though, in order to discuss the results conveniently and make this paper easy to read, we would refer to them as quitter and relapser groups in the following context.

### MRI Acquisition Parameters

All the scans were performed on a 3.0T GE MR scanner in the Department of Radiology, Second Affiliated Hospital of Zhejiang University. Ear plugs and foam pads were used to reduce noise and head motion. High resolution axial T1- and T2-weighted anatomic images were first obtained to exclude any dormant neurological diseases. Diffusion tensor imaging was acquired using a SE-EPI sequence. The scan parameters were as follows: repetition time = 12,000 ms; echo time = 105 ms; acquisition matrix = 128 × 128; field of view = 240 mm × 240 mm; slice thickness = 3 mm; 37 contiguous axial slices. Diffusion images were acquired from 31 gradient directions (*b* = 1000 s/mm^2^) with an acquisition without diffusion weighting (*b* = 0). Several other sequences were also acquired on the participants but were not included in this study. A total scan for each subject lasted about 40 min.

### Data Processing

Diffusion-weighted images were analyzed using the brain fMRI software library (FSL, version 4.1.0^1^). The original data were first converted to compressed FSL NIFTI format. The DWI images with different diffusion gradients were visually inspected to ensure there were no abrupt head motions during the scan. Then skull stripping was performed using the brain extraction toolbox. After eddy current correction, diffusion tensors were reconstructed and diffusion parameters were calculated. We visually inspected the resulting FA images to ensure that major fiber tracts were displayed correctly. Then the TBSS procedure was applied. After aligning the individual FA images to the standard space template using non-linear registration, the mean FA image was calculated and compressed to form a mean skeleton representing topological features of all tracts derived from the whole group. An FA threshold of 0.3 was set to remove trivial tracts. Finally, each subject’s aligned FA images were projected onto the fiber skeleton template to perform statistical analysis. Mean diffusivity (MD) was also normalized to the skeleton using TBSS.

### Statistical Analysis

Age and education were compared among the three groups using one-way analysis of variance. Smoking indices and cognitive scales were compared between the two outcome groups using independent two-sample *t*-test. The image data, including FA and MD skeletonized images, were analyzed using one-way analysis of variance by the FSL randomize procedure with 5000 permutations. Age and education were included as covariates to adjust group comparisons for their potential influences. To reduce the family wise error rate induced by multiple comparisons, we employed a cluster-based thresholding method (*p* < 0.01 with 100 contiguous voxels). To label fiber locations, the coordinates of the clusters’ peak points were referenced by the Johns Hopkins University white matter atlas and MRI Atlas of Human White Matter, 2nd edition (Oishi et al., 2010). After these steps, we extracted average FA values from the clusters showing main effects among the three groups. A *post hoc* test was performed to examine where the difference came from and was corrected using Bonferroni method. To explore white matter integrity changes in all smokers, we also pooled the quitters and relapsers together and compared their FA and MD maps with non-smokers.

We also explored the relationship between diffusion parameters and smoking behaviors in all smokers. Specifically, linear regression models were used with regional FA as the dependent measure, with smoking variables and age as predictors.

## Results

### Demographic Characteristics

The three groups were not significantly different in age or education level (**Table 1**). The quitter group and relapser group were not significantly different in number of years smoked over lifetime (NYS), number of cigarettes smoked per day (CSPD), pack years, or FTND, suggesting that the two groups have similar cigarette consumption and nicotine addiction severity. Their scores on MMSE, HRSD, and HAMA did not have differences.

**Table 1:** Demographic characteristics.

	Non-smoker (*n* = 37)	Quitters (*n* = 28)	Relapser (*n* = 38)	*P*
Age (y)	38.21 ± 8.49 (26∼56)	38.96 ± 6.77 (27∼54)	38.61 ± 6.81 (28∼51)	0.913
Education (y)	14.94 ± 4.02	13.36 ± 2.61	13.71 ± 2.77	0.102
Years smoked over life (y)	–	17.29 ± 6.32	19.61 ± 6.39	0.148
Cigarettes smoked per day	–	24.11 ± 10.55	22.24 ± 9.7	0.458
Pack-year	–	21.61 ± 14.96	21.91 ± 12.23	0.929
FTND	–	5.32 ± 2.34	5.13 ± 2.17	0.735
MMSE	–	29.07 ± 1.63	29.13 ± 1.02	0.855
HRSD	–	1.82 ± 4.71	1.13 ± 1.82	0.412
HAMA	–	1.89 ± 5.10	0.63 ± 1.51	0.154

### Imaging Results

**FIGURE 1:**
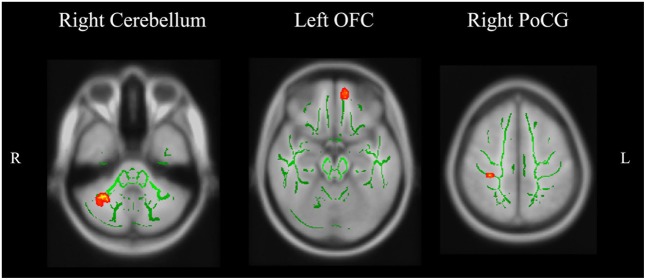
Brain regions showing significant differences of FA among the three groups.

**FIGURE 2:**
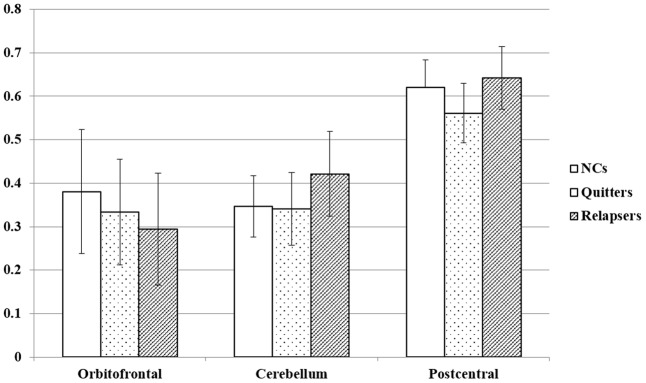
Average FA values of the three clusters in the three groups.

**FIGURE 3:**
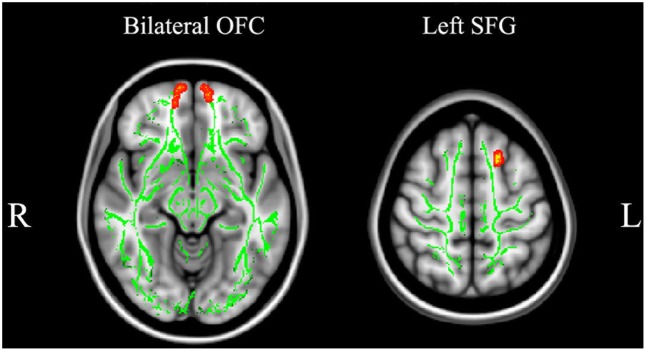
Pooled smokers had lower FA in bilateral orbitofrontal gyrus and left superior frontal gyrus than non-smokers.

As shown in **Figure 1**, main effects of group in FA appear in the left orbitofrontal gyrus (OFC), the right cerebellum, and the right postcentral gyrus (**Table 2**). The relapsers had significantly lower FA in the left OFC (*p* = 0.01, corrected, **Figure 2**) than non-smokers, and higher FA in the right cerebellum than non-smokers (*p* = 10^-4^, corrected) and quitters (*p* = 0.001, corrected). Meanwhile, the quitters had significantly lower FA in the right postcentral gyrus compared to non-smokers (*p* = 0.001, corrected) and relapsers (*p* = 10^-5^, corrected). Compared to non-smokers (**Figure 3** and **Table 2**), pooled smokers had lower FA in bilateral OFC and left superior frontal gyrus (SFG). We did not find any significant differences in MD among the three groups.

**Table 2:** Brain areas where FA showed significant differences among the three groups.

Nearest gray matter	Voxels	Peak p	MNI coordinates	

**ANOVA results**				***Post hoc* significant pairs**
Right cerebellum	172	0.002	37 -54 -38	Relapsers > Non-smokers
				Relapsers > Quitters
Right postcentral gyrus	164	0.002	31 -32 52	Quitters < Non-smokers
				Quitters < Relapsers
Left orbitofrontal gyrus	156	0.002	-7 50 -20	Relapsers < Non-smokers

**Non-smoker > Pooled smokers**				

Left superior frontal gyrus	114	0.001	-17 18 52	–
Right orbitofrontal gyrus	257	0.002	11 55 -25	–
Left orbitofrontal gyrus	147	0.002	-15 58 -14	–

Furthermore, linear regression analysis revealed that FA in the left orbitofrontal gyrus was predicted by behavioral scores (model *p* = 0.026). The NYS (*p* = 0.024) and CSPD (*p* = 0.007) were significant predictors. Partial correlation (r) between orbitofrontal FA and NYS/CSPD was -0.286 and -0.340, respectively. FA in other areas could not be predicted by behavioral scores.

## Discussion

In the present study, we demonstrated the white matter alterations in smokers and investigated its relationship with smoking behaviors and future cessation outcomes. We found that pooled smokers had smaller FA than non-smokers in bilateral OFC and left SFG. Also, the relapsers and quitters had different FA alteration patterns compared to non-smokers. Among the differential brain regions, the left orbitofrontal FA was associated with smoking behaviors in all smokers. Furthermore, the relapsers had higher FA in right cerebellum and right post central gyrus than quitters. These findings indicate that white matter integrity is altered in smokers, and is related to smoking behaviors and cessation outcomes.

Firstly, white matter integrity is altered in smokers. The pooled smokers had decreased FA in bilateral OFC. The OFC’s function includes integrating and evaluating multimodal information and making decisions. Damage to the OFC may induce enhanced stress reactivity, an inability to suppress emotions (Bechara et al., 2000), increased motivation to procure drugs (Rolls, 2000) and decreased ability to inhibit drug-cue related behaviors (Volkow and Fowler, 2000), which may lead to substance addiction. While OFC abnormalities have been frequently linked to addiction behaviors, there have been no reports of OFC white matter alterations in smokers. Instead, several previous studies found gray matter deficits and functional impairments in the OFC of smokers. For example, cortical thinning in the OFC and increased volume of the caudate have been reported in smokers (Dom et al., 2005; Kühn et al., 2010), suggesting the contribution of fronto-striatal damage to nicotine addiction. Smokers showed abnormal brain activation in response to smoking-related stimuli than neutral stimuli in the OFC, amygdala and some other brain areas (Dinh-Williams et al., 2014). In addition to group differences, we also found that OFC FA was associated with NYS/CSPD, providing evidence for further understanding OFC’s role in nicotine addiction.

Besides, the pooled smokers also showed lower FA in the left SFG, which is located at the dorsolateral prefrontal cortex (dlPFC). The dlPFC is the center for advanced cognitive functions such as working memory, planning and inhibition (Barbey et al., 2013; Kaller et al., 2013). Several studies in smokers suggest that the dlPFC is hyper-active during the abstinence period, and may contribute to craving symptoms (Mcclernon et al., 2005, 2009). Suppressing dlPFC activity using transcranial magnetic stimulation may help smokers remain abstinence (Rose et al., 2011; Trojak et al., 2015). In contrast, morphometry studies found decreased gray matter density (Brody et al., 2004) and volume (Fritz et al., 2014) in the dlPFC. It is possible that dlPFC structural deficits may damage its cognitive functions, and lead to uncontrolled response to smoking cues. Though, previous DTI studies have not reported white matter disruption in this area. Our findings support that dlPFC deficit is associated with nicotine addiction.

Compared to non-smokers, the relapsers (but not quitters) had higher FA in the cerebellum. Although the cerebellum is traditionally considered to be important for motor control, recent functional imaging studies suggest that it is also involved in cognitive functions (Stoodley, 2012). Specifically, the cerebellum may be involved in nicotine addiction by playing a role in the formation of habitual behaviors. Repeated drug use may re-organize related cortical-cerebellum circuitry, strengthening drug-related memories, expectations, and motivation behaviors (Miquel et al., 2009). Cerebellum gray matter reduction has been consistently reported in smokers (Kühn et al., 2012; Franklin et al., 2014). Additionally, functional imaging studies usually reveal increased activity in this area. Nicotine treatment may increase smokers’ cerebellar activation during a finger tapping task (Wylie et al., 2013). Chu et al. (2013) found increased resting-state activity in the posterior lobe of the cerebellum in smokers. It has been suggested that those increased activities might be related to a Pavlovian associative mechanism (Bonson et al., 2002) and drug craving (Schneider et al., 2001). Therefore, chronic smokers may have increased fiber connectivity due to long-term potentiation effects.

The fact that FA can be both decreased and increased in different brain areas of smokers suggests that cigarette smoking has complicated influences on the human brain. Several studies also revealed differential modulatory effects of smoking on white matter (Lin et al., 2013; Yu et al., 2016). When discussing these results, many factors need to be taken into consideration. On one hand, long-term smoking can cause increased brain responses and strengthened fiber connections, leading to compulsive and habitual behaviors. This effect is especially prominent in adolescent smokers whose brains are still under continuous development (Jacobsen et al., 2007). Several studies of young smokers also demonstrate elevated fiber connectivity (Yu et al., 2016). On the other hand, nicotine is detrimental to the human cardiovascular system. Long-term cigarette smoking may cause severe damage to brain vessels and lead to neuronal death. This effect is prominent in elder smokers, who showed greater age-related white matter volume loss and fiber integrity disruption (Lin et al., 2013). For middle-aged smokers, previous findings are not consistent. Both increased and decreased FA have been reported in different studies (Paul et al., 2008; Kochunov et al., 2013; Savjani et al., 2014; Durazzo et al., 2017). Therefore, here the complex FA alterations may be the result of both an enhancement and a degeneration effect.

Secondly, the two outcome groups had different fiber integrity. Specifically, relapsers had higher cerebellum and postcentral FA than quitters. These findings suggest that, although the two outcome groups had similar nicotine addiction severity, the relapsers might have strengthened neural circuitry related to smoking behaviors. As discussed above, the cerebellum is related to the formation of habitual behaviors, and may contribute to drug craving. While the role of postcentral gyrus in nicotine addiction is still unknown, a study found that activation in the sensory-motor regions in response to drug-related cues could predict drug relapse behaviors (Kosten et al., 2006). Considering the general functions of these two structures and the related evidence, we suggest that the higher FA here may indicate the formation of habitual/automatic smoking behaviors that promotes smoking relapse.

Previously, other modalities of MRI have been used to reveal the neural mechanisms of smoking cessation. For example, amygdala response to smoking cues (Janes et al., 2010; Perkins, 2011) or cessation messages (Chu et al., 2013) in smokers was predictive of cessation outcome. Similarly, brain activation to emotional pictures was related to cessation success rate (Versace et al., 2014). It has been suggested that compared to relapsers, successful smoking quitters have significantly higher GM volume in the left putamen and right occipital lobe, but lower GM volume in the bilateral hippocampus and right cuneus (Froeliger et al., 2010). Before this study, whether white matter alterations are related to cessation outcomes was not sufficiently studied. Here, we provide evidence that FA is associated with different cessation outcomes. As diffusion tensor imaging reflects the white matter integrity, it has a unique advantage compared to other imaging modalities. As discussed above, while these brain areas are known to be related to cognitive/habitual processing, how they are related to Varenicline treatment is still unclear. Varenicline displays full agonism on α7 nicotinic acetylcholine receptors and is a partial agonist on some other subtypes (Niaura et al., 2006). It may take effect through downregulating brain activities in certain brain regions (Franklin et al., 2011), but enhanced fiber connections may keep the drug from modulating the related circuitry, leading to higher relapse rate.

This study has several limitations. The first is the modest sample size. The second is that we had not collected detailed clinical measures from non-smokers. Although we had ruled out any potential neuropsychiatric conditions and major diseases that could affect the brain, a quantified record would certainly be more accurate to reflect group characteristics. The third is that all our subjects were male. As female smokers are scarce in China, it is difficult to recruit enough subjects to achieve a balance between genders and we decided to enroll only male subjects. Therefore, our results and inferences may not be applicable to female smokers. In addition, further studies are still needed to demonstrate the relationship between longitudinal brain changes and cessation outcomes.

## Conclusion

We found white matter integrity alterations in the OFC and SFG of smokers. Besides, relapsers and quitters had different baseline white matter integrity in the right cerebellum and postcentral gyrus. These results suggest that white matter integrity alterations may be the underlying reason for the relatively low efficacy of smoking cessation treatments. We believe that future studies utilizing machine learning on white matter characteristics of the smokers may have great potential to predict smoking cessation outcomes.

## Author Contributions

PH, YY, and MZ were responsible for the study concept and design. CW and WQ contributed to the acquisition of imaging data. PH, ZS, and HZ assisted with data analysis and interpretation of findings. PH drafted the manuscript. YY and MZ provided critical revision of the manuscript for important intellectual content. All authors critically reviewed content and approved final version for publication.

## Conflict of Interest Statement

The authors declare that the research was conducted in the absence of any commercial or financial relationships that could be construed as a potential conflict of interest. The reviewer YZ and handling Editor declared their shared affiliation.

## Footnotes

1 http://www.fmrib.ox.ac.uk/fsl
